# Diabetes, gender and deterioration in estimated glomerular filtration rate in patients with chronic heart failure: Ten-year prospective cohort study

**DOI:** 10.1177/1479164120984433

**Published:** 2021-02-15

**Authors:** Jessica Kearney, Michael Drozd, Andrew MN Walker, Thomas A Slater, Sam Straw, John Gierula, Maria Paton, Judith Lowry, Charlotte Cole, Klaus K Witte, Richard M Cubbon, Mark T Kearney

**Affiliations:** Leeds Institute for Cardiovascular and Metabolic Medicine, University of Leeds, Leeds, UK

**Keywords:** Diabetes, heart failure, eGFR, ACE-I

## Abstract

**Introduction:**

We aimed to evaluate the relationship between temporal changes in renal function and long-term mortality in patients with heart failure with reduced ejection fraction (HFrEF) and identify correlates of deteriorating renal function.

**Methods:**

A total of 381 patients with HFrEF enrolled in a prospective cohort study between 2006–2014 had eGFR measured at initial visit and at 1 year. Baseline characteristics were used in a multivariate analysis to establish variables that predict deterioration in eGFR. Follow-up data were used to assess whether declining eGFR was related to outcomes.

**Results:**

Patients were grouped into tertiles based on percentage change in eGFR. In a multivariate logistic regression analysis, male sex was associated with a 1.77-fold ([95% CI 1.01–2.89]; *p* = 0.045) and diabetes a 1.66-fold ([95% CI 1.02–2.70]; *p* = 0.041) greater risk of a decline in eGFR compared to those with stable/improving eGFR. Declining eGFR was associated with a 1.4-fold greater risk of death over 10 years ([95% CI 1.08–1.86]; *p* = 0.01) and a 3.12-fold ([1.44–6.75]; *p* = 0.004) greater risk of death at 1 year from second eGFR measurement.

**Conclusions:**

In patients with HFrEF diabetes and male sex are independent predictors of a decline in eGFR at 1 year. A decline eGFR over 1 year is associated with higher long-term all-cause mortality.

## Introduction

Amongst the multiple co-morbidities frequently associated with chronic HF due to reduced ejection fraction (HFrEF) the most common are diabetes mellitus (DM) in around 24%^1^ and chronic kidney disease (CKD) in 40%–50%.^2^ Due to their high prevalence and causal interrelationship, they commonly co-exist, presenting not only therapeutic challenges but also synergistically adversely affecting patient-orientated outcomes including mortality.^2–4^

Whilst previous meta-analysis demonstrated that both baseline renal impairment and worsening renal function (WRF) (defined as either decline in eGFR or rise in creatinine) are associated with a worse outcome in HFrEF patients^2^ and that the key predictors of WRF include baseline CKD, hypertension, diabetes, age and diuretic use, several of the studies included had relatively short follow up and were before the era of modern heart failure therapy. Moreover, there remains no specific evidence-based, effective treatment of patients with HFrEF experiencing WRF. Identifying those patients most at risk could allow for personalised monitoring, medication adjustment and, in due course, help explore the potential benefits of novel strategies and therapeutic targets.

## Aims of the study

In the present analysis, we used data from those patients in a prospective cohort study of ambulant patients with HFrEF who had both baseline and 1-year follow-up eGFR measurement in the outpatient setting. We aimed to evaluate the degree and predictors of change in renal function over time, and to explore the relationship between WRF and mortality in a well-phenotyped contemporary cohort. In particular, we hypothesised that diabetes is a risk factor for worsening renal function in patients with HFrEF.

## Methods

We conducted a prospective cohort study with the pre-defined aim of investigating prognostic factors in patients with HFrEF receiving contemporary evidence-based medical and device therapies.^5,6^ Consecutive unselected patients were recruited in specialist heart failure clinics in four UK hospitals and provided written informed consent. Inclusion in the study required stable clinical signs and symptoms of CHF (chronic heart failure) for at least 3 months and left ventricular ejection fraction ⩽45% on transthoracic echocardiography. This analysis is restricted to an unselected subgroup of consecutive patients (*n* = 385) recruited between January 2006 to January 2009 (visit 1) who had a follow-up assessment at 1-year (visit 2) with paired assessment of renal function. Patients (*n* = 4) receiving renal replacement therapy were excluded, for a final study population of 381. The Leeds West Research Ethics Committee gave ethical approval (07/Q1205/17) and the study complies with the principles of the Declaration of Helsinki.

As previously described,^6^ baseline characteristics collected at study recruitment including demographics, past medical history, electrocardiography, cardiac imaging and treatment. Symptomatic status was defined based upon reported symptoms using the New York Heart Association (NYHA) Functional Classification. Venous blood was collected for measurement of full blood count, electrolytes and assessment of renal function. Estimated glomerular filtration rate (eGFR) was calculated using the Modification of Diet in Renal Disease method.^7^ Two-dimensional transthoracic echocardiography was performed according to the American Society of Echocardiography recommendations. Resting heart rate and other electrocardiographic variables were measured using 12-lead resting electrocardiograms. Prescribed medical therapy was recorded and the total daily doses of ACEi, β-blockers and loop diuretics were expressed relative to the maximal licenced dose of ramipril, bisoprolol and furosemide, respectively, as we have previously published.^6^ All patients were registered with the UK Office of Population Censuses and Surveys, which provided details of death; follow-up censorship occurred on 8 November 2018.

### Statistical analysis

After confirming normality of distribution, continuous descriptive group data are presented as the mean ± standard deviation (SD) whilst percentage (number) are provided for categorical data. Groups were compared using Student *t*-tests or ANOVA for normally distributed continuous data, Mann-Whitney *U*-tests or Kruskal-Wallis H-tests for non-normally distributed continuous data, and Pearson’s chi-square statistic tests for categorical data.

Change in eGFR from visit 1 to visit 2 was grouped into tertiles. To explore predictors of change in eGFR, binary logistic regression was used with decline in eGFR as a binary dependent variable (decline vs stable or improved eGFR). Kaplan Meier curves were used to plot survival and compared using log-rank tests. Adjusted survival analyses used Cox regression analysis. Statistical significance was accepted as *p* < 0.05. All statistical analyses were performed with SPSS version 21 software (SPSS Inc., Chicago, IL, USA).

## Results

**Figure 1.**
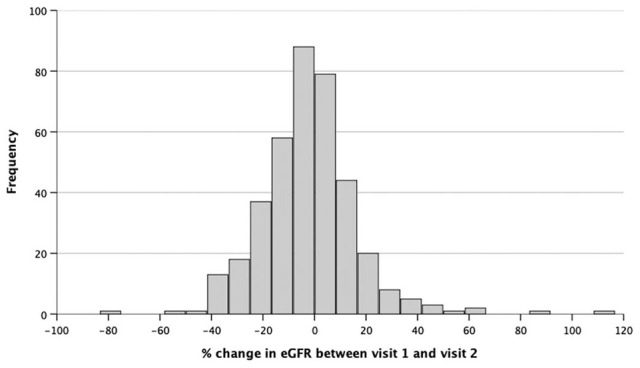
Distribution of percentage change in eGFR from recruitment and 1-year follow-up measurement.

Baseline characteristics of the entire cohort are presented in Table 1. The distribution of percentage change in eGFR between recruitment and 1-year is shown in Figure 1. The mean (SD) of absolute change in eGFR between recruitment and follow up attendances was −1.62 (SD 9.6) ml/kg. This translated into a mean percentage change in eGFR over the 12 months of follow up of −2.11% (SD 19.13%). The mean change in eGFR was −2.76% (SD 19.46) for men and −0.20% (SD 18.11) for women (*p* = 0.24).

**Table 1.** Characteristics of patients according to tertiles of change in eGFR from recruitment to 1-year follow-up measurement.

Characteristic	Total (*n* = 381)	Tertile			*p* value
		Decline (T1) (−79.04 to −8.49; *n* = 127)	Stable (T2) (−8.48 to +3.69; *n* = 127)	Improving (T3) (+3.73 to 112.06; *n* = 127)	
Age, *y*	66.5 ± 12.1	67.7 ± 11.5	65.9 ± 12.3	65.9 ± 12.4	0.40
Male	284 (74.5)	103 (81.1)	84 (66.1)	97 (76.4)	0.02
Heart rate, bpm	73.3 ± 14.5	72.3 ± 15	72.9 ± 14.8	74.8 ± 13.7	0.55
Systolic BP, mmHg	121.8 ± 22	123.9 ± 23.2	121.5 ± 21.3	120.1 ± 21.5	0.36
Diastolic BP, mmHg	71.7 ± 12.4	72.4 ± 13.1	71 ± 12.8	71.7 ± 11.2	0.66
LVEF (%)	30.8 ± 9.3	31.5 ± 9	30.6 ± 9.4	30.5 ± 9.5	0.65
Hb, g/dL	13.9 ± 1.8	13.8 ± 1.9	13.9 ± 1.6	14 ± 1.9	0.70
Baseline eGFR, mL/kg per min	53.4 ± 15.6	54.8 ± 17.2	55.2 ± 13.7	50.3 ± 15.4	0.022
Follow-up eGFR, mL/kg per min	51.8 ± 16.6	43.4 ± 15.4	53.9 ± 13.3	58.1 ± 17.2	<0.001
Ramipril dose, mg/d	5.1 ± 3.5	5.2 ± 3.6	4.9 ± 3.5	5.1 ± 3.6	0.73
Bisoprolol dose, mg/d	3.3 ± 3	3.1 ± 2.9	3.1 ± 3	3.8 ± 3	0.11
Furosemide dose, mg/d	54.1 ± 51.1	56.9 ± 47.9	52.9 ± 58.8	52.5 ± 46	0.76
MRA prescription	158 (41.7)	49 (38.6)	50 (39.7)	59 (46.8)	0.35
Diabetes	92 (24.1)	39 (30.7)	26 (20.5)	27 (21.3)	0.11
Ischaemic aetiology	242 (63.5)	86 (67.7)	75 (59.1)	81 (63.8)	0.36
NYHA Class					0.36
1	81 (21.3)	25 (19.7)	29 (22.8)	27 (21.3)	
2	166 (43.6)	55 (43.3)	62 (48.8)	49 (38.6)	
3	129 (33.9)	44 (34.6)	36 (28.3)	49 (38.6)	
4	5 (1.3)	3 (2.4)	0 (0)	2 (1.6)	

We initially divided the cohort into tertiles (T1-T3) based on percentage change in eGFR, which approximately defined groups with deteriorating, stable or improving eGFR. Those with declining renal function received comparable heart failure therapy and diuretic doses, although there were significant differences in male sex, baseline eGFR and follow-up eGFR between the tertiles. A total of 92 (24.1%) patients had diabetes with a mean HbA_1c_ of 57.5 mmol/mol (SD 17.7 mmol/mol).

To explore the predictors of a decline in eGFR (T1), we next grouped T2 and T3 together to represent a stable or improving change in eGFR (Table 2). Follow-up eGFR was significantly lower in those with declining eGFR (43.4 ± 15.4 mL/kg per min) compared to those with stable or improving eGFR (56.0 ± 15.5 mL/kg per min) *p* < 0.001. Patients with declining eGFR were more likely to be male and have diabetes compared to those with stable or improving eGFR. In univariate logistic regression analysis of these variables, only male sex (OR 1.73 CI 1.03–2.91; *p* = 0.039) and diabetes (OR 1.68 CI 1.04–2.73; *p* = 0.035) were significantly associated with risk of decline in eGFR (Table 3). Furthermore, ramipril dose (OR 1.02 CI 1.02; *p* = 0.63) and Bisoprolol dose (OR 0.97 CI 0.90–1.04; *p* = 0.33) were not associated with risk of decline in eGFR.

**Table 2.** Characteristics of patients according to decline (T1) compared to stable or improving (T2 and T3) change in eGFR from recruitment to 1-year follow-up measurement.

Characteristic	Decline (T1) (−79.04 to −8.49; *n* = 127)	Stable or improving (T2 and T3) (−8.48 to +122.06; *n* = 254)	*p* value
Age, *y*	67.7 ± 11.5	65.9 ± 12.3	0.17
Male	103 (81.1)	191 (71.3)	**0.038**
Heart rate, bpm	72.3 ± 15	73.8 ± 14.3	0.46
Systolic BP, mmHg	123.9 ± 23.2	120.8 ± 21.4	0.20
Diastolic BP, mmHg	72.4 ± 13.1	71.3 ± 12	0.44
LVEF (%)	31.5 ± 9	30.5 ± 9.4	0.35
Hb, g/dL	13.8 ± 1.9	13.9 ± 1.7	0.64
eGFR, mL/kg per min	54.8 ± 17.2	52.8 ± 14.8	0.27
Follow-up eGFR, mL/kg per min	43.4 ± 15.4	56.0 ± 15.5	**<0.001**
Ramipril dose, mg/d	5.2 ± 3.6	5 ± 3.5	0.63
Bisoprolol dose, mg/d	3.1 ± 2.9	3.4 ± 3	0.33
Furosemide dose, mg/d	56.9 ± 47.9	52.7 ± 52.7	0.44
MRA prescription	49 (38.6)	109 (43.3)	0.38
Diabetes	39 (30.7)	53 (20.9)	**0.034**
Ischaemic aetiology	86 (67.7)	156 (61.4)	0.23
NYHA Class			0.60
1	25 (19.7)	56 (22.0)	
2	55 (43.3)	111 (43.7)	
3	44 (34.6)	85 (33.5)	
4	3 (2.4)	2 (0.8)	

Bold values denote statistical significance (*p* < 0.05).

**Table 3.** Univariate predictors of decline in eGFR from recruitment to 1-year follow-up measurement.

Characteristic	OR	95% CI	*p* value
Age, *y*	1.01	0.99–1.03	0.18
Male	1.73	1.03–2.91	**0.039**
Heart rate, bpm	0.99	0.97–1.01	0.45
Systolic BP, mmHg	1.01	1.00–1.01	0.19
Diastolic BP, mmHg	1.01	0.99–1.03	0.43
LVEF (%)	1.01	0.99–1.04	0.35
Hb, g/dL	0.97	0.86–1.09	0.63
Baseline eGFR, mL/kg per min	1.01	1.00–1.02	0.24
Ramipril dose, mg/d	1.02	0.96–1.08	0.63
Bisoprolol dose, mg/d	0.97	0.90–1.04	0.33
Furosemide dose, mg/d	1.00	0.98–1.01	0.46
MRA prescription	0.83	0.53–1.27	0.38
Diabetes	1.68	1.04–2.73	**0.035**
Ischaemic	1.32	0.84–2.07	0.23
NYHA Class			
1	reference		
2	1.11	0.63–1.97	0.72
3	1.16	0.64–2.10	0.63
4	3.36	0.53–21.4	0.20

Bold values denote statistical significance (*p* < 0.05).

In a multivariate logistic regression analysis of male sex and diabetes, male sex was associated with a 1.77-fold ([95% CI 1.01–2.89]; *p* = 0.045) and diabetes a 1.66-fold ([95% CI 1.02–2.70]; *p* = 0.041) greater risk of decline in eGFR (Table 4). When age was added into the model, diabetes as a predictor lost statistical significance (*p* = 0.052).

**Table 4.** Multivariate predictors of decline in eGFR from recruitment to 1-year follow-up measurement.

Model	Characteristic	OR	95% CI	*p* value
Age, male	Age	1.01	1.00–1.03	0.16
	Male	1.75	1.04–2.95	**0.036**
Male, Diabetes	Male	1.71	1.01–2.89	0.045
	Diabetes	1.66	1.02–2.70	0.041
Age, Male, Diabetes	Age	1.01	0.99–1.03	0.21
	Male	1.73	1.02–2.92	**0.041**
	Diabetes	1.62	1.00–2.64	0.052

Bold values denote statistical significance (*p* < 0.05).

**Figure 2.**
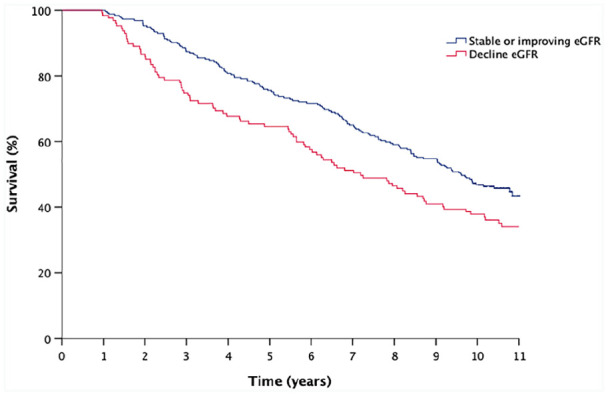
Kaplan-Meier curves illustrating survival comparing percentage change in eGFR from recruitment to 1-year follow-up measurement.

We next explored long-term survival and present 2893 patient-years of follow-up with median follow-up of 9 years (IQR 4.3–10.6 years). Kaplan-Meier survival curves (Figure 2) revealed significantly different survival between those with decline in eGFR compared to stable or improving eGFR (log-rank *p* = 0.011). In a Cox regression analysis, declining eGFR was associated with a 1.4-fold greater risk of death ([95% CI 1.08–1.86]; *p* = 0.01) compared to stable or improving eGFR. When age, sex and diabetes were added to the model (Table 5), the hazard ratio reduced slightly and lost statistical significance (HR 1.2 CI 0.99–1.71; *p* = 0.061).

**Table 5.** Crude and adjusted all-cause mortality of decline in eGFR from recruitment to 1-year follow-up measurement.

	All-cause mortality		
	HR	95% CI	*p*
Unadjusted			
Decline in eGFR	1.42	(1.08–1.86)	**0.011**
Adjusted for gender			
Decline in eGFR	1.38	(1.05–1.81)	**0.020**
Adjusted for age, gender			
Decline in eGFR	1.35	(1.03–1.77)	**0.029**
Adjusted for age, gender, diabetes			
Decline in eGFR	1.26	(0.96–1.65)	0.10

Bold values denote statistical significance (*p* < 0.05).

We reviewed the risk of death at 1 year following second eGFR measurement. Declining eGFR was associated with a 3.12-fold increase in risk of death at 1 year ([1.44–6.75]; *p* = 0.004) compared with stable or improving eGFR. When adjusted for age, gender and diabetes the risk of death was 2.77 greater ([1.24–6.17]; *p* = 0.013). 13.9% of patients showing a decline in eGFR are dead at 1 year compared with 4.7% without a decline in eGFR.

In those with a low baseline eGFR, small absolute changes in eGFR could result in disproportionately higher percentage changes in eGFR. We therefore conducted a sensitivity analysis excluding people with eGFR <30 (*n* = 25) and found broadly similar findings (Supplemental Material).

## Discussion

Our prospective, cohort study followed patients with HFrEF in the outpatient setting. Our most important findings are:

- Patients with HFrEF that have a 1-year decline in eGFR have a 3.12 increased risk of all-cause mortality at 1 year.
- Diabetes and male sex are independent predictors of a decline in eGFR.
- ACE-inhibitor, Beta-blocker and diuretic doses were not associated with a decline in eGFR.

Previous studies have shown that patients with CHF have an increased risk of developing CKD^7^ and an association between CHF and a decline in renal function in those with and without pre-existing CKD has been observed.^2,8,9^ eGFR normally declines at 1 to 2 mL/min/1.73 m^2^ per year in aging populations and Khan et al. demonstrated that 36% of CHF patients had a decrease in eGFR of >5 mL/min per 1.73 m^2^ per year.^9^ The average decline in eGFR in our study was 1.62 per year.

Whilst other studies have observed an association between a decline in renal function and all-cause mortality following hospital admission or post myocardial infarction,^10,11^ there are few that study patients with HFrEF in the outpatient setting. Khan et al. performed a retrospective analysis of the Studies of Left Ventricular Dysfunction (SOLVD) studies of 6640 patients with HFrEF in the ambulatory setting over an average of 2.6 years where the rate of decline of eGFR was associated with mortality.^9^ However, the data were collected before the era of beta-blockers and device therapies in CHF.^12^ De Silva et al., collected prospective data on 1216 ambulatory HFrEF patients. A rise in serum creatinine >26.5 micromol/L, was observed in 13% of patients over 6 months, and baseline CKD and WRF were associated with higher mortality.^13^ Our study has confirmed the association between decline in eGFR and mortality, however, we have examined a well-characterised patient group on modern heart failure therapies with a median follow-up is 9 years (max follow-up 12.8 years).

The association between CHF and CKD, or WRF, is probably multifactorial.^14^ There are several mechanisms by which WRF could cause or exacerbate CHF including haemodynamic changes,^15^ sympathetic overactivity,^16^ inflammation^17^ and the induction of apoptosis by uremic toxins.^18^ On the other hand, CHF has been hypothesised to cause WRF via low cardiac output and increased renal venous pressure, neurohormonal mechanisms such as sympathetic nervous system and renin-angiotensin system (RAAS) activation, and inflammatory activation.^8^

Medications used to treat HFrEF also have an impact on renal function. RAAS inhibitors are known to cause an initial WRF and there is conflicting evidence as to the long-term impact of this. Schmidt et al. demonstrated that a rise in creatinine after initiating ACE-I or Angiotensin Receptor Blockers (ARBs) was associated with adverse cardiac outcome and death and that this risk correlated with the level of rise in creatinine.^19^ However, a meta-analysis showed that the outcomes of CHF patients with WRF after treatment with placebo were shown to be worse than WRF induced by RAAS inhibitors.^20^ It is thought that, despite WRF, there is net benefit from treatment.^21^ Importantly, our study demonstrated no association with ACEi dose and WRF. Similarly, beta-blocker dose was also not associated with a fall in eGFR and previous studies have not seen a deterioration in renal function with beta-blocker use.^22, 23^

CHF and CKD share risk factors, including diabetes, hypertension, age, race and gender. Diabetes is the most common cause of CKD in the UK and patients with diabetes have a two-fold risk of developing HF.^24^ Our group has previously demonstrated that diabetes doubles the risk of death in patients with CHF despite contemporary treatment.^25^ In the present study, diabetes was observed to be a predictor of eGFR decline. Male sex was also a predictor for decline in eGFR. However, other studies have demonstrated female sex as a risk factor for WRF in CHF patients.^9,10^ Males have a greater age-related decline in eGFR and those with CKD have a greater chance of progression to end-stage renal failure and death compared with females.^26,27^ Age has previously been highlighted as a risk factor; but this was not seen in our study.^9,28^

### Study strengths and limitations

Our study used data from a well characterised cohort with a median follow up of 9 years. The dataset included patients with HFrEF on contemporary therapies in the outpatient setting, making the results applicable to modern CHF patients. However, several limitations must be acknowledged. The study was an observational study and so we are unable to comment on causality and we also cannot describe a mechanism for the link between male sex, diabetes and worsening renal function. In addition, our dataset excludes patients with CHF and preserved ejection fraction, so we cannot generalise the outcomes to this patient group.

### Clinical implications of the present study

The present study has highlighted the need for therapeutics that reduce the decline in eGFR in HFrEF. The rate of decline of renal function has been shown to be associated with mortality in patients with CKD^29^ and recognising this decline early can improve outcomes for CKD patients via education, avoidance of nephrotoxics and modification of risk factors such as hypertension, glycaemic control and iron deficiency anaemia.^30^ Similarly, recognising HFrEF patients with high risk of WRF could enable interventions to slow progression and reduce the observed increased mortality. Those with higher rates of decline may require more regular monitoring of their renal function. Men have also been highlighted as being at increased risk, and an awareness of this could encourage more intense monitoring and education.

We have demonstrated that DM is an independent risk factor for the decline in eGFR in patients with HFrEF, emphasising the need for medications that effectively target DM in this patient group. SGLT2 inhibitors are a class of drugs that are used in the treatment of type 2 DM and were first approved for use in the UK in 2012. The EMPA-REG OUTCOME trial sought to establish the long-term cardiovascular safety, and potential benefits, from the SGLT2 inhibitor empagliflozin.^31^ This randomised control trial studied patients with type 2 DM and high cardiovascular risk. Patients treated with empagliflozin were seen to have reduced rates of heart failure related hospitalisation, cardiovascular death and all-causes mortality versus those treat with placebo. Similarly, the CANVAS trial saw reduced risk of cardiovascular deaths, MI and stroke with the SGTL2- inhibitor Canagliflozin versus placebo.^32^

In addition to the cardiovascular benefits with SGLT2 inhibitor treatment, improved renal outcomes have been observed. The DAPA-CKD trial studied the effect of dapagliflozin on renal outcomes in patients with CKD. Compared with placebo treatment, patients treated with dapagliflozin had reduced risk of a decline in eGFR, end-stage kidney disease and death from renal or cardiovascular causes compared with placebo. This effect was observed in patients both with and without type 2 DM.^33^ A meta-analysis of the DAPA-HF and EMPEROR-Reduced trials demonstrated that SGLT2 inhibitors improve renal outcomes, cardiovascular and all-cause mortality in HFrEF patients.^34^ SGLT2 inhibitors were not standard therapy for patients with type 2 DM and HFrEF at the time of our data collection. However, they are now licensed for use the treatment of HFrEF in patients with and without diabetes. We have highlighted DM as a risk factor for declining eGFR in our patient group and quantified the substantial increased mortality that accompanies this, our data therefore adds evidence for the potential benefits from SGLT2 inhibitor treatment in patients with HFrEF.

Another key finding of our study is that ACEi doses were not associated with a decline in eGFR. The guidelines for the use of ACEi in HFrEF are based on large-scale randomised control trials where high target doses are set.^13^ A recent meta-analysis has demonstrated the reduction in all-cause mortality and HF hospitalisations associated with higher versus lower doses of ACEi.^35^ A further meta-analysis did not see the same reduction in mortality but did observe reduced risk of HF worsening with higher doses.^36^ However, a significant concern for many healthcare professionals is that neurohumoral modification, in particular ACEi, leads to a disadvantageous decline in eGFR and as a result optimal doses of these agents are often not achieved.^37^ In our study, ramipril dose was not associated with a decline in eGFR. This is an important finding and should empower physicians to titrate ACEi to the maximum tolerated dose.

## Conclusion

We have demonstrated in a well-characterised cohort of patients with HFrEF that decline in eGFR is associated with increased all-cause mortality. We have highlighted that male gender and diabetes are risk factors for a decline in eGFR whilst ACEi and beta-blocker dose are not associated with a decline.

## Supplemental Material

sj-docx-1-dvr-10.1177_1479164120984433 – Supplemental material for Diabetes, gender and deterioration in estimated glomerular filtration rate in patients with chronic heart failure: Ten-year prospective cohort study Click here for additional data file. Supplemental material, sj-docx-1-dvr-10.1177_1479164120984433 for Diabetes, gender and deterioration in estimated glomerular filtration rate in patients with chronic heart failure: Ten-year prospective cohort study by Jessica Kearney, Michael Drozd, Andrew MN Walker, Thomas A Slater, Sam Straw, John Gierula, Maria Paton, Judith Lowry, Charlotte Cole, Klaus K Witte, Richard M Cubbon and Mark T Kearney in Diabetes & Vascular Disease Research
