# Supplementary material for: Diabetes, gender and deterioration in estimated glomerular filtration rate in patients with chronic heart failure: Ten-year prospective cohort study
Source: Diab Vasc Dis Res. 2021 Feb 15;18(1):1479164120984433. doi: 10.1177/1479164120984433 (PMC8481744; doi:10.1177/1479164120984433)
Supplement: sj-docx-1-dvr-10.1177_1479164120984433 – Supplemental material for Diabetes, gender and deterioration in estimated glomerular filtration rate in patients with chronic heart failure: Ten-year prospective cohort study [file sj-docx-1-dvr-10.1177_1479164120984433.docx]

**Supplementary Data**

Excluding patients with baseline eGFR <30 mL/kg per min (*n*=25), cohort size *n*=356

**Supplementary table 1** Univariate predictors of decline in eGFR from recruitment to 1-year follow-up (excluding patients with baseline eGFR <30 mL/kg per min)

| **Characteristic** | **OR** | **95% CI** | ***P value*** |
| --- | --- | --- | --- |
| Age, y | 1.01 | 0.99-1.03 | 0.28 |
| Male | 1.85 | 1.06-3.24 | 0.03 |
| Heart rate, bpm | 0.99 | 0.98-1.01 | 0.52 |
| Systolic BP, mmHg | 1.01 | 1.00-1.02 | 0.16 |
| Diastolic BP, mmHg | 1.01 | 0.99-1.03 | 0.31 |
| LVEF (%) | 1.01 | 0.99-1.04 | 0.35 |
| Hb, g/dL | 1.01 | 0.89-1.15 | 0.91 |
| Baseline eGFR﻿, mL/kg per min | 1.01 | 1.00-1.03 | 0.09 |
| Ramipril dose, mg/d | 1.01 | 0.95-1.08 | 0.69 |
| Bisoprolol dose, mg/d | 0.96 | 0.89-1.04 | 0.29 |
| Furosemide dose, mg/d | 1.00 | 1.00-1.01 | 0.30 |
| MRA prescription | 0.84 | 0.54-1.32 | 0.46 |
| Diabetes | 1.87 | 1.12-3.1 | 0.016 |
| Ischaemic | 1.3 | 0.82-2.05 | 0.28 |
| NYHA Class |  |  |  |
| 1 | reference |  |  |
| 2 | 1.08 | 0.60-1.93 | 0.80 |
| 3 | 1.07 | 0.58-1.98 | 0.82 |
| 4 | 3.3 | 0.52-21.00 | 0.21 |

**Supplementary table 2** Multivariate predictors of decline in eGFR from recruitment to 1-year follow-up (excluding patients with baseline eGFR <30 mL/kg per min)

| **Model** | **Characteristic** | **OR** | **95% CI** | ***P value*** |
| --- | --- | --- | --- | --- |
| Age, male | Age | 1.01 | 0.99-1.03 | 0.28 |
|  | Male | 1.85 | 1.06-3.24 | 0.031 |
|  |  |  |  |  |
| Male, Diabetes | Male | 1.82 | 1.04-3.19 | 0.038 |
|  | Diabetes | 1.83 | 1.10-3.06 | 0.020 |
|  |  |  |  |  |
| Age, Male, Diabetes | Age | 1.01 | 0.99-1.03 | 0.39 |
|  | Male | 1.82 | 1.04-3.20 | 0.037 |
|  | Diabetes | 1.79 | 1.07-3.00 | 0.026 |

**Supplementary table 3** Crude and adjusted all-cause risk of mortality in patients with decline in eGFR from recruitment to 1-year follow-up (excluding patients with baseline eGFR <30 mL/kg per min)

|  | All-cause mortality | | | |
| --- | --- | --- | --- | --- |
|  | HR | 95% CI | p |  |
|  |  |  |  |  |
| Unadjusted |  |  |  |  |
| Decline in eGFR | 1.39 | 1.05-1.85 | 0.022 |  |
|  |  |  |  |  |
| Adjusted for gender |  |  |  |  |
| Decline in eGFR | 1.34 | 1.01-1.78 | 0.046 |  |
|  |  |  |  |  |
| Adjusted for age, gender |  |  |  |  |
| Decline in eGFR | 1.34 | 1.01-1.78 | 0.045 |  |
|  |  |  |  |  |
| Adjusted for age, gender, diabetes |  |  |  |  |
| Decline in eGFR | 1.23 | 0.92-1.65 | 0.157 |  |
|  |  |  |  |  |
